# The Evolutionary Concept of “Preadaptation” Applied to Cognitive Neurosciences

**DOI:** 10.3389/fnins.2016.00103

**Published:** 2016-03-17

**Authors:** Alfredo Ardila

**Affiliations:** Communication Sciences and Disorders, Florida International UniversityMiami, FL, USA

**Keywords:** preadaptation, complex cognition, cognitive evolution, cognitive neurosciences

The term preadaptation in evolution refers to a large change in function accomplished with little or no change in structure (Ridley, 2004). That is, preadaptation refers to the possibility of a characteristic to adopt a new biological function without evolutionary modification. The idea that the function of a trait might shift during its evolutionary history was initially developed by Darwin (1859). This phenomenon is usually known as “preadaptation.” However, since this term may suggest teleology, it has been proposed to be replaced by the term “exaptation” (Gould and Vrba, 1982). I shall refer, however, to preadaptation because it is the most frequently used name.

Preadaptation can refer both to anatomical or behavioral characteristics. An anatomical example frequently mentioned, pertains to bird feathers. At first, birds' feathers were essentially for heat insulation rather than an adaptation for flight; this initial evolutionary purpose for warmth represents a preadaptation for flight. The following is a behavioral example of preadaptation: subdominant wolves lick the mouths of alpha wolves as a sign of submissiveness; this behavior can be related to the wolf pups licking the faces of adults to encourage them to regurgitate food (preadaptation).

*Homo sapiens* has existed for about 150,000 years without evident neurological changes (Carroll, 2003). We can assume that 150,000 years ago *Homo sapiens* possessed certain fundamental cognitive abilities required to survive. During these 150,000 years, however, significant changes in cognition have occurred, without evident changes in his brain morphology. These changes in cognition include, but are not limited to, the development of a complex grammatical language, reading, writing, calculation abilities, and meta-cognitive executive functions (Ardila, 2004, 2008, 2011, submitted). These new cognitive abilities are based on those fundamental intellectual abilities that existed in *Homo sapiens* 150,000 years ago, which represent the pre-adaptations for these new cognitive strategies: grammatical language, reading, writing, etc.

There have been very few attempts to relate current cognitive abilities with their preadaptation. Varney (1984) proposed a model for Kanji reading applicable in a non-Japanese population of aphasics. Aphasic subjects were required to match animals with their footprints, a type of ideogram “reading” requiring no special training. The author suggested that matching animals with their footprints represents a preadaptation of Kanji word reading. He found that all aphasic patients with defects in footprint reading were also impaired in letter recognition or pantomime recognition, but some aphasics with defects in word reading performed normally in animal footprint reading.

I will propose some probable preadaptations for several forms of complex cognition: language grammar, calculation abilities, reading, writing, and metacognitive executive functions.

First of all, grammar or morphosyntax refers to the set of rules governing the construction of words or morphology, as well as the construction of sentences or syntax, in a particular language. No question, grammar represents the most advanced and complex aspect of human language (Chomsky, 1965; Bickerton, 2007). Grammar requires the use of verbs, and verbs represent actions; consequently, those brain areas involved in recognizing actions may become adapted to process grammar. They are the preadaptations of grammatical language. As a matter of fact, it has been observed that, (a) the classical Broca's area or Brodmann area 44 becomes activated during observation of expressive gestures and motor acts (Lotze et al., 2006); and (b) the activation of Brodmann area 44 during different linguistic tasks results in a co-activation of some occipital lobe areas (Bernal et al., 2015). These two observations suggest that the perception of actions represent a preadaptation of grammatical language. Grammar quite likely developed based on the ability to recognize actions; and both language grammar and recognition of actions are depending upon the same brain systems.

It is worth noting that several lines of research suggest that the appearance of grammar involved more than one evolutionary step. I do not deny this point; the case that I make is just that action recognition is the most fundamental preadaptation supporting its emergence.

Finger knowledge probably represents a preadaptation of the ability to calculate. Counting most likely began with finger sequencing (Ardila, 2010), as it is observed in children; furthermore, the use of a finger sequencing in counting may explain the 10-base found in most numerical systems. From the clinical perspective, there is a strong relationship between numerical knowledge and finger recognition; both are impaired in cases of left posterior parietal damage, in the so-called angular or Gerstmann's syndrome (Gerstmann, 1940), suggesting a common neurological substrate. On the other hand, there exists a strong relationship between acalculia understood as an acquired impairment in calculation abilities and right-left discrimination disorders. That means, calculation abilities are directly related with finger recognition and right left discrimination; in cases of brain pathology, they appear in a single cortical syndrome, known as angular or Gerstmann's syndrome. It has been proposed that this syndrome is defined as a defect in verbally mediated spatial operations (Ardila, 2014). Consequently, finger knowledge and right-left discrimination, which represents the use of spatial relations in language, probably are preadaptations of calculation abilities.

Reading represents a particular ability to associate phonemes, the auditory components of language, and their visual representations. There are some fundamental forms of acquired disturbances in reading that indicate the preadaptations required to read. Classically, two different types of alexia have been recognized (Dejerine, 1891, 1892): (1) pure or agnosic alexia, or without agraphia alexia, observed in cases of left occipital pathology, and usually interpreted as a visuo-perceptual disturbance; simply speaking, a visual agnosia, probably a particular subtype of simultanagnosia (Farah, 2004); (2) alexia with agraphia or temporal-parietal alexia, which is characterized by an inability to associate the sounds of the language with their visual representations; that means, this particular type of alexia represents a disturbance in cross-modal associations (Benson, 1979). Alexia can also be observed in cases of right hemisphere damage, due to the contralateral neglect; it is the so-called spatial or visuospatial alexia (Hecaen, 1972; Ardila and Rosselli, 1994). Consequently, reading is primarily based on the following three abilities or preadaptations: visual perception, cross-modal associations, and spatial perception.

These skills required to read and write are only partially coincidental. It has been proposed that writing began with the ability to draw; drawing is a constructive ability that progressively became associated with some particular meanings expressed in oral language; that is, writing is based on some cross-modal associations. Furthermore, some specific standardized movements or praxic abilities are required to write the letters and words (Ardila, 2004). In consequence, at least the following fundamental abilities or preadaptations are required to write words: (1) constructive abilities are required to “draw” letters and words in a particular space; (2) cross-modal association; that is, the ability to associate the “draws” with oral language; and (3) some praxic abilities are needed to make standardized movements to write the letters and words. Of course, writing beyond individual letters and words, such as writing sentences and texts, also require some additional abilities, for instance, verbal memory and executive functions.

“Metacognitive executive functions” include problem solving, abstracting, planning, strategy development and implementation, working memory, and similar complex cognitive abilities (Ardila, 2008). It has been difficult to find the common factor underlying these complex intellectual functions. Fuster (1997, 2002) stated that the most general executive function of the dorsolateral prefrontal cortex, or in other words, the common factor of metacognitive executive functions, is the temporal organization of goal-directed actions in the domains of behavior, cognition, and language. If the temporal organization of behavior and cognition represent the core executive functions factor, the question becomes, where behavioral temporality comes from? Temporality means “before” and “after,” that is, something that changes, or develops or moves, that is, an action. Simply speaking, the “perception of actions” would represent a single preadaptation both for grammatical language and for meta-cognitive executive functions. As a matter of fact, it has been proposed that both, grammatical language and metacognitive executive functions appeared simultaneously in human evolution (Ardila, 2008, 2015). It is evident that whereas language vocabulary contains a categorization or conceptualization system, language grammar contains a thinking or reasoning strategy.

Table 1 summarizes the probable preadaptations for language grammar, calculation abilities, reading, writing, and metacognitive executive functions.

**Table 1:** **Some contemporary cognitive abilities and their probable preadaptations**.

**Cognitive ability**	**Preadaptation(s)**
Grammatical language	Perception of actions
Calculation abilities	Finger knowledge Spatial relations in language
Reading	Visual perception Cross-modal associations Spatial perception
Writing	Constructive abilities Cross-modal associations Praxic abilities
Meta-cognitive executive functions	Perception of actions (grammatical language)

In summary, contemporary complex cognition, such as written language, meta-cognitive executive functions, and others, represent a further evolution of some basic abilities or preadaptations existing 150,000 years ago when *Homo sapiens* first appeared.

## Author contributions

The author confirms being the sole contributor of this work and approved it for publication.

### Conflict of interest statement

The author declares that the research was conducted in the absence of any commercial or financial relationships that could be construed as a potential conflict of interest.

## References

[B1] Ardila A. (2004). There is not any specific brain area for writing: from cave painting to computers. Intern. J. Psychol. 39, 61–67. 10.1080/00207590344000295

[B2] Ardila A. (2008). On the evolutionary origins of executive functions. Brain Cogn. 68, 92–99. 10.1016/j.bandc.2008.03.003 18397818

[B3] Ardila A. (2010). On the evolution of calculation abilities. Front. Evolut. Neurosci. 2:7. 10.3389/fnevo.2010.00007 20725520 PMC2912036

[B4] Ardila A. (2011). There are two different language systems in the brain. J. Behav. Brain Sci. 1, 23–36. 10.4236/jbbs.2011.12005

[B5] Ardila A. (2014). A proposed reinterpretation of gerstmann's syndrome. Arch. Clin. Neuropsychol. 29, 828–833. 10.1093/arclin/acu056 25377466

[B6] Ardila A. (2015). A proposed neurological interpretation of language evolution. Behav. Neurol. 2015, 16. 10.1155/2015/872487 26124540 PMC4466361

[B7] Ardila A. Rosselli M. (1994). Spatial alexia. Intern. J. Neurosci. 76, 49–59. 10.3109/00207459408985991 7960468

[B8] Benson D. F. (1979). Aphasia, alexia, and agraphia. London: Churchill Livingstone.

[B9] Bernal B. Ardila A. Rosselli M. (2015). Broca's area network in language function: a pooling-data connectivity study. Front. Psychol. 6:687. 10.3389/fpsyg.2015.00687 26074842 PMC4440904

[B10] Bickerton D. (2007). Language evolution: a brief guide for linguists. Lingua 117, 510–526. 10.1016/j.lingua.2005.02.006

[B11] Carroll S. B. (2003). Genetics and the making of *Homo sapiens*. Nature 422, 849–857. 10.1038/nature01495 12712196

[B12] Chomsky N. (1965). Aspects of the Theory of Syntax. Cambridge, MA: MIT Press.

[B13] Darwin C. (1859). On the Origin of Species. London: John Murray.

[B14] Dejerine J. (1891). Sur un cas de cécité verbale avec agraphie suivi d'autopsie. Compt. Rend. Soc. Biol. 3, 197–201.

[B15] Dejerine J. (1892). Contribution à l'étude anatomo-pathologique et clinique des différents variétés de cécité verbale. Compt. Rend. Soc. Biol. 4, 61-90.

[B16] Farah M. J. (2004). Visual Agnosia. Cambridge, MA: MIT press.

[B17] Fuster J. M. (1997). The Prefrontal Cortex—Anatomy Physiology, and Neuropsychology of the Frontal Lobe. Philadelphia: Lippincott-Raven.

[B18] Fuster J. M. (2002). Frontal lobe and cognitive development. J. Neurocytol. 31, 373–385. 10.1023/A:1024190429920 12815254

[B19] Gerstmann J. (1940). Syndrome of finger agnosia, disorientation for right and left, agraphia and acalculia: local diagnostic value. Arch. Neurol. Psychiatry 44, 398–408. 10.1001/archneurpsyc.1940.02280080158009

[B20] Gould S. J. Vrba E. S. (1982). Exaptation—a missing term in the science of form. Paleobiology 8, 4–15. 10.1017/S0094837300004310

[B21] Hecaen H. (1972). Introduction a la neuropsychologie. Paris: Larousse

[B22] Lotze M. Heymans U. Birbaumer N. Veit R. Erb M. Flor H.. (2006). Differential cerebral activation during observation of expressive gestures and motor acts. Neuropsychologia 44, 1787–1795. 10.1016/j.neuropsychologia.2006.03.016 16730755

[B23] Ridley M. (2004). Evolution. Hoboken, NJ: Wiley-Blackwell.

[B24] Varney N. R. (1984). Alexia for ideograms: Implications for kanji alexia. Cortex 20, 535–542. 10.1016/S0010-9452(84)80056-8 6518794

